# Changes of brain functional network in Alzheimer’s disease and frontotemporal dementia: a graph-theoretic analysis

**DOI:** 10.1186/s12868-024-00877-w

**Published:** 2024-07-04

**Authors:** Shijing Wu, Ping Zhan, Guojing Wang, Xiaohua Yu, Hongyun Liu, Weidong Wang

**Affiliations:** 1 Medical Innovation Research Division, Chinese PLA General Hospital, Beijing, 100853 China; 2 Key Laboratory of Biomedical Engineering and Translational Medicine, Ministry of Industry and Information Technology, Beijing, 100853 China

**Keywords:** Alzheimer’s disease, Frontotemporal dementia, EEG, Functional connectivity, Graph-theoretic analysis

## Abstract

**Background:**

Alzheimer’s disease (AD) and frontotemporal dementia (FTD) are the two most common neurodegenerative dementias, presenting with similar clinical features that challenge accurate diagnosis. Despite extensive research, the underlying pathophysiological mechanisms remain unclear, and effective treatments are limited. This study aims to investigate the alterations in brain network connectivity associated with AD and FTD to enhance our understanding of their pathophysiology and establish a scientific foundation for their diagnosis and treatment.

**Methods:**

We analyzed preprocessed electroencephalogram (EEG) data from the OpenNeuro public dataset, comprising 36 patients with AD, 23 patients with FTD, and 29 healthy controls (HC). Participants were in a resting state with eyes closed. We estimated the average functional connectivity using the Phase Lag Index (PLI) for lower frequencies (delta and theta) and the Amplitude Envelope Correlation with leakage correction (AEC-c) for higher frequencies (alpha, beta, and gamma). Graph theory was applied to calculate topological parameters, including mean node degree, clustering coefficient, characteristic path length, global and local efficiency. A permutation test was then utilized to assess changes in brain network connectivity in AD and FTD based on these parameters.

**Results:**

Both AD and FTD patients showed increased mean PLI values in the theta frequency band, along with increases in average node degree, clustering coefficient, global efficiency, and local efficiency. Conversely, mean AEC-c values in the alpha frequency band were notably diminished, which was accompanied by decreases average node degree, clustering coefficient, global efficiency, and local efficiency. Furthermore, AD patients in the occipital region showed an increase in theta band node degree and decreased alpha band clustering coefficient and local efficiency, a pattern not observed in FTD.

**Conclusions:**

Our findings reveal distinct abnormalities in the functional network topology and connectivity in AD and FTD, which may contribute to a better understanding of the pathophysiological mechanisms of these diseases. Specifically, patients with AD demonstrated a more widespread change in functional connectivity, while those with FTD retained connectivity in the occipital lobe. These observations could provide valuable insights for developing electrophysiological markers to differentiate between the two diseases.

## Introduction

Both Alzheimer’s disease (AD) and frontotemporal dementia (FTD) are highly prevalent neurodegenerative diseases, accounting for 60-80% and 10% of all dementia cases respectively [1, 2]. However, the pathological mechanisms of these two dementias still remain unclear, and currently, there is no cure for either type, this lack of a cure increases the psychological and economic burden on patients and their families [3]. With the rapid development of brain science, research on brain network connectivity offers a deeper comprehension of the brain’s structure, function, and pathological mechanisms, this understanding, in turn, can aid in the prevention, diagnosis, and treatment of brain diseases [4].

Functional brain network is a powerful tool for researching brain connectivity, which is sensitive to neurodegeneration. It can be applied to understand the brain’s functions and physiopathological mechanisms [4–6]. A functional brain network is typically derived from time series data, describing statistical patterns of dynamic interactions between brain regions. With the development of modern medical imaging technology, the sources for these time series can include electroencephalogram (EEG), magnetoencephalography (MEG) or functional magnetic resonance imaging (fMRI). Compared to MEG and fMRI, EEG is a relatively cost-effective and highly available technique with high temporal resolution [7, 8] and is widely used in functional connectivity studies of various neurological and psychiatric diseases, including schizophrenia [9, 10], autism spectrum disorder [11, 12] and epilepsy [13, 14].

Functional brain network may provide a macroscale scaffolding to explain pathophysiological abnormalities [15, 16]. Further, graph-theoretical approaches can quantify these abnormalities [17]. Recent studies of neurological and mental disorders have demonstrated that graph-theoretical approaches can reveal meaningful information about the topological architecture of the human brain network. This may offer novel insights into the biological mechanisms underlying human cognition, as well as health and disease [4, 18–21].

AD is often considered to be a ‘disconnection syndrome’ [22]. A plethora of EEG studies have reported a reduction in the strength of functional connectivity between different brain regions in AD patients [23–26]. Several studies have indicated that these patients exhibit a more random pattern of functional connectivity compared to healthy controls [27, 28]. However, research into brain network connectivity in FTD remains relatively sparse. A resting-state EEG study revealed that patients with FTD exhibited abnormal microstates linked to the activation of the frontal lobe [29]. Yu et al. discovered that AD patients showed significantly reduced brain functional connectivity in the delta and alpha frequency bands compared to those with FTD [30]. De Haan et al. reported a decreased clustering coefficient in the lower alpha and beta bands for AD patients compared to controls, but no significant differences were found in FTD patients in these measures [31]. Bonanni et al. observed greater strength in functional network connectivity at the prodromal stage of dementia in both AD and FTD patients [32]. Franciotti et al. reported a derangement in the cortical network modularity, observed only in FTD patients [33]. Furthermore, an fMRI study indicated that AD patients exhibited lower mean nodal strength, lower local efficiency, and longer mean path length in the parietal lobe compared to FTD patients [5].

Despite these endeavors in exploring the abnormalities of brain network connectivity in AD and FTD, a consensus has not yet been established. The pathophysiological mechanisms underlying these two types of dementia have not been fully elucidated, and no reliable biomarkers have been delineated. Therefore, further investigation into the brain network abnormalities in AD and FTD is necessary. In this study, we aimed to investigate the abnormalities of brain functional networks in AD and FTD using EEG recordings and graph theory analysis. First, the Phase Lag Index (PLI) was measured for the low-frequency bands (delta and theta) and the Amplitude Envelope Correlation with leakage correction (AEC-c) was measured for the high-frequency bands (alpha, beta, and gamma) across the three groups (AD, FTD, and HC). Then, we constructed a binary brain network and calculated general topological parameters, including mean node degree, clustering coefficient, characteristic path length, global efficiency, and local efficiency. Moreover, we divided brain regions based on electrode position and computed the mean node degree, clustering coefficient, and local efficiency for different brain regions within the theta and alpha frequency band. Our observations regarding the functional network topology and connectivity abnormalities in AD and FTD may contribute to a better understanding of the pathophysiological mechanisms of these conditions.

## Methods

### Data source

In this work, we used the OpenNeuro public dataset, which includes preprocessed resting seated state-closed eyes EEG recordings of AD (*n* = 36), FTD (*n* = 23) and HC (*n* = 29). The initial diagnosis for AD and FTD patients was performed according to the criteria provided by the “Diagnostic and Statistical Manual of Mental Disorders,” 3rd edition, revised (DSM-III-R), the 4th edition (DSM-IV), and the International Classification of Diseases, 10th revision (ICD-10) [34]. Additionally, the criteria from the National Institute of Neurological Disorders and Stroke-Alzheimer’s Disease and Related Disorders Association [35] were also considered. For recording, a Nihon Kohden EEG 2100 clinical device was used, with 19 scalp electrodes (Fp1, Fp2, F7, F3, Fz, F4, F8, T3, C3, Cz, C4, T4, T5, P3, Pz, P4, T6, O1, and O2) according to the 10–20 international system and 2 reference electrodes (A1 and A2) placed on the mastoids for impedance checks. The channel impedances were maintained at less than 5 KΩ, and the sampling frequency was set at 500 Hz.

For preprocessing, the original EEG signals were conducted in EEGLAB Matlab software. First, a Butterworth bandpass filter (0.5–45 Hz) was applied and the signals were re-referenced to A1-A2. Then, the Artifact Subspace Reconstruction routine which is an EEG artifact correction method was applied to remove bad data periods. Next, the Independent Component Analysis (ICA) method was performed, transforming the EEG signals to 19 ICA components. ICA components were then recognized as “eye artifacts” or “jaw artifacts” by the automatic classification routine “ICLabel” and automatically rejected.

### Construction of binary undirected brain network

We constructed a binary undirected brain network to investigate the abnormalities in brain connectivity associated with AD and FTD. Determining the nodes and edges within this network was essential. In this study, we defined the 19 EEG electrodes as network nodes and utilized functional connectivity measures to characterize the network edges. Before calculating functional connectivity, we filtered the EEG signals into delta (0.5–4 Hz), theta(4–8 Hz), alpha (8–13 Hz), beta (13–30 Hz), and gamma (30–45 Hz) frequency bands. We then segmented the continuous EEG trials into epochs of 12.288 s with a 50% overlap [36]. In the delta and theta frequency bands, we analyzed the strength of functional connectivity for all EEG epochs of each subject using the PLI [37] in the HERMES toolbox [38]. For the alpha, beta, and gamma frequency bands, we evaluated connectivity strength by measuring the AEC-c [39] in the Brainstorm toolbox [40].

The PLI is an index that measures the degree of phase synchronization between two signals, based on the asymmetry of the distribution of instantaneous phase differences. It is insensitive to shared signals with zero phase lag:

$$PLI = \left| {\left\langle {sign\left[ {sin(\Delta \phi ({t_k}))} \right]} \right\rangle } \right|$$

where $\varDelta \phi \left({t}_{k}\right)$ is the phase difference at time point ${t}_{k}$ between two time series, calculated for all time-points per epoch; $sign$ stands for signum function; < > denotes the mean value; and ⎢⎢indicates the absolute value. PLI values range between 0 and 1, where 0 indicates no (nonzero-lag) coupling and 1 refers to perfect (nonzero-lag) phase locking. The phases were estimated using the Hilbert transform.

The amplitude envelope correlation (AEC) [41] is an amplitude-based metric which estimates the coupling between two time series by estimating the Pearson’s correlation between the envelopes of the amplitudes of these time series.

$$AEC=corr ({h}_{x}\left(t\right), {h}_{y}\left(t\right))$$

where ${h}_{x}\left(t\right)$ and ${h}_{y}\left(t\right)$ are the envelopes of time series x and y, respectively, corr is the Pearson’s correlation coefficient. The envelopes were estimated using the Hilbert transform. The AEC-c is an improved measure of AEC that first applies pair-wise symmetric orthogonalization (linear regression analysis) to time series data to remove zero-lag correlations caused by volume conduction, and then estimates the linear correlation between the envelopes of band-pass filtered signals.

To compare the brain network connectivity among the three groups, we averaged the PLI/AEC-c matrices for each group and visualized the brain network connectivity using the BrainNet Viewer toolbox [42].

Meanwhile, we constructed binary undirected brain networks based on a threshold. The threshold was selected as the maximum value at which no isolated nodes appear in the network [43]. The threshold values for the theta and alpha frequency bands were 0.0462 and 0.0890, respectively.

### Brain network analysis

In this work, 5 common graph theory parameters were computed using the Brain Connectivity Toolbox [44] to analyze the topology and attributes of the brain network. The node degree ${K}_{i}$ is the number of links connected to the node *i*. The larger the node degree, the more important the node is in the whole network.

$${K}_{i}=\sum _{j}{a}_{ij}$$

The mean node degree $K$ is the sum of the node degrees of all nodes divided by the number of nodes.

The clustering coefficient reflects the level of interconnection between the adjacent nodes of a node. The clustering coefficient ${C}_{i}$ of a node *i* is defined as the ratio of the actual number of connected edges to the maximum number of possible connected edges.

$${C}_{i}=\frac{2{E}_{i}}{{K}_{i}({K}_{i}-1)}$$

where ${K}_{i}$ is the node degree of node $i$, and ${E}_{i}$ is the actual number of edges between neighbors of node $i$. The clustering coefficient $C$ of a network is the sum of the clustering coefficient of all nodes divided by the number of nodes.

The characteristic path length $L$ indicates the overall efficiency of information integration between different brain regions. It is the average of all shortest path lengths between all pairs of nodes.

$$L =\frac{1}{M(M-1)}\sum _{i\ne j}{l}_{ij}$$

where $M$ is the number of nodes, and ${l}_{ij}$ is the shortest path length between nodes $i$ and $j$.

The global efficiency ${E}_{glob}$ measures the global transmission capacity of a network. It is usually defined as the inverse of all shortest path lengths in a network.

$${E}_{glob}=\frac{1}{M(M-1)}\sum _{i\ne j}\frac{1}{{l}_{ij}}$$

The local efficiency ${E}_{i}$ describes the ability of a network to transmit local information, which is calculated in a way similar to global efficiency, except that it is calculated at the level of individual nodes rather than at the level of the entire network.

$$E\left(i\right)=\frac{1}{{M}_{{G}_{i}}({M}_{{G}_{i}}-1)}\sum _{j\ne k\in {G}_{i}}\frac{1}{{l}_{jk}}$$

where ${G}_{i}$ refers to the subgraph formed by the neighbors of node $i$, and ${l}_{jk}$ represents the shortest path length between nodes $j$ and $k$. The local efficiency ${E}_{loc}$ of the network is the average of the local efficiencies of all nodes.

$${E}_{loc}=\frac{1}{M}\sum E\left(i\right)$$

### Statistical analysis

Statistical analyses for baseline group characteristics were performed using IBM SPSS Statistics 22.0 software (IBM SPSS Statistics, Chicago, IL, United States). Age, gender, and Mini-Mental State Examination (MMSE) scores were compared among the three groups (AD, FTD, and HC) using the non-parametric Kruskal-Wallis one-way ANOVA test, followed by a post hoc Bonferroni correction for multiple comparisons.

For brain network connectivity values and network topological parameters, we employed permutation testing to compare the statistical differences among the three groups. If the results were significantly different, permutation tests were performed on pairwise group comparisons (AD vs. HC, FTD vs. HC, and AD vs. FTD), and the p-values for all the pairwise multiple comparisons were corrected using Bonferroni correction.

## Results

### Subject characteristics

The main demographic and clinical characteristics of the AD, HC, and FTD are listed in Table 1. No significant difference was found in the level of age and gender between all group combinations. The MMSE score was lower in AD than that in the FTD, and the FTD group showed a lower MMSE score than the HC group.

**Table 1:** Demographic and clinical characteristics in patients with AD, HC, and patients with FTD. In the Gender column, F indicates female and M indicates male. MMSE stands for Mini-Mental State Examination. IQR means Interquartile Range. The p-value refers to the result of the non-parametric Kruskal-Wallis one-way ANOVA test, followed by post hoc pairwise comparisons (Bonferroni-corrected for multiple comparisons)

	AD	HC	FTD	*p*: AD vs. HC	*p*: FTD vs. HC	*p*: AD vs. FTD
**N**	36	29	23	−	−	−
**Age** **mean (SD)**	66.4(7.9)	67.9(5.4)	63.6(8.2)	> 0.05	> 0.05	> 0.05
**Gender, F/M**	24/12	11/18	9/14	0.066	1.000	0.121
**MMSE** **mean (SD)**	17.75(4.5)	30(0)	22.17(8.2)	< 0.001	< 0.001	0.020
**Disease duration** **median (IQR)**	25(4.5)	-	25(4.5)	-	-	-

### Mean PLI/AEC-c values in five frequency bands

**Fig. 1:**
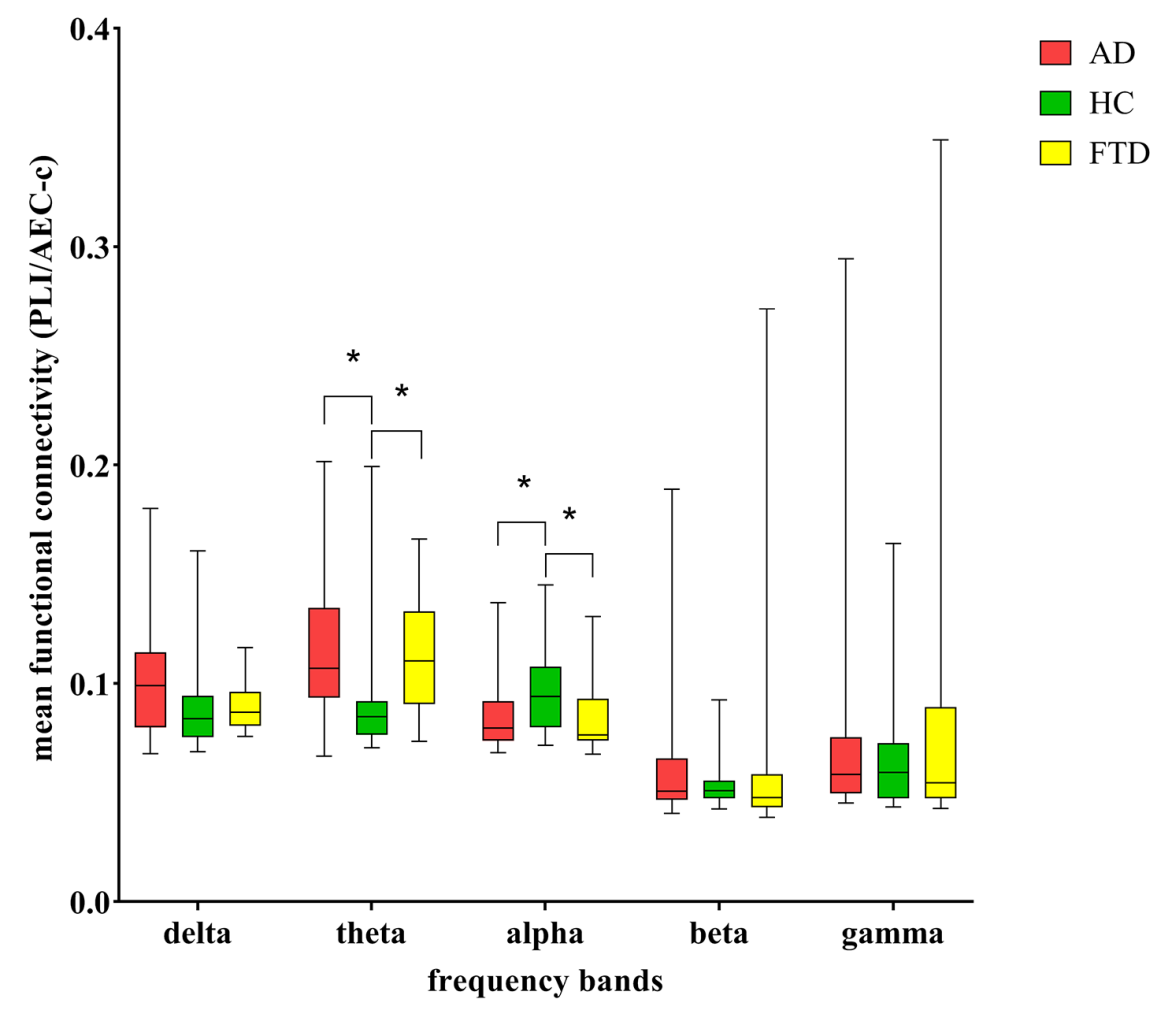
The average PLI values of the delta and theta bands and the average AEC-c values of the alpha, beta, and gamma bands. The significant differences are denoted by the asterisk (corrected *p* < 0.05)

The average PLI values of the delta and theta bands and the average AEC-c values of the alpha, beta, and gamma bands are shown in Fig. 1. In the theta band, the mean PLI values for both AD patiens and FTD patients are significantly lower than that of HC (AD vs. HC: *p* = 0.021, FTD vs. HC: *p* = 0.048, AD vs. FTD: *p* = 1.000). On the contrary, the mean AEC-c values for both AD patients and FTD patients are significantly higher than that of HC (AD vs. HC: *p* = 0.016, FTD vs. HC: *p* = 0.043, AD vs. FTD: *p* = 1.000) in the alpha band. No significant differences in mean functional connectivity values were found between the three groups in the delta, beta, and gamma bands.

**Fig. 2:**
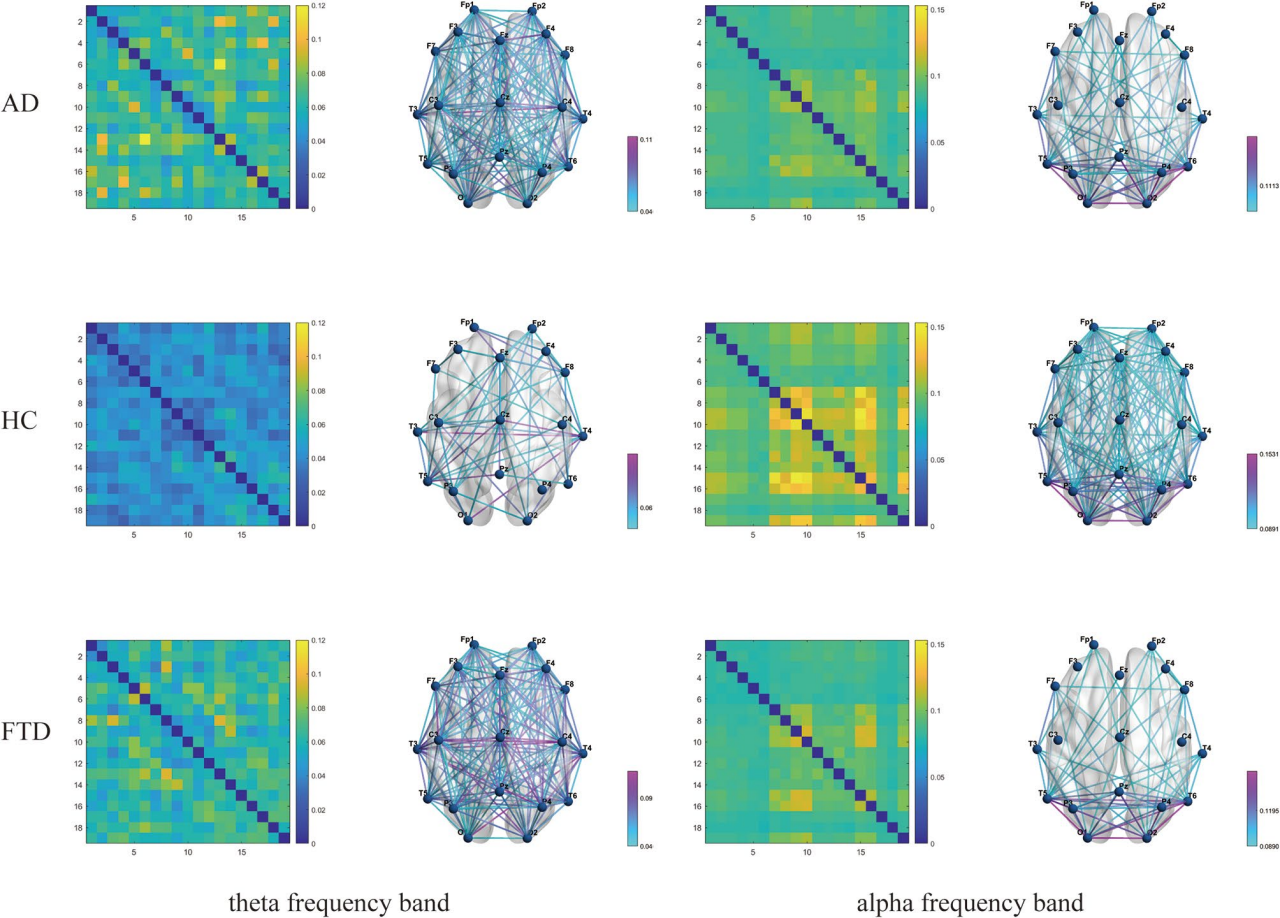
Mean PLI matrices in the theta band and mean AEC-c matrices in the alpha band and and their brain network connectivity graphs

The averaged PLI matrices of the three groups in the theta band and the averaged AEC-c matrices in the alpha band were subsequently converted into brain network graphs, as shown in Fig. 2. We observed a significant increase in brain network connectivity in AD and FTD patients at the theta frequency band (AD vs. HC: *p* = 0.021, FTD vs. HC: *p* = 0.048, AD vs. FTD: *p* = 1.000). In contrast, there is significant weakness in brain network connectivity in the alpha band (AD vs. HC: *p* = 0.016, FTD vs. HC: *p* = 0.043, AD vs. FTD: *p* = 1.000).

### Brain network topological parameters

**Fig. 3:**
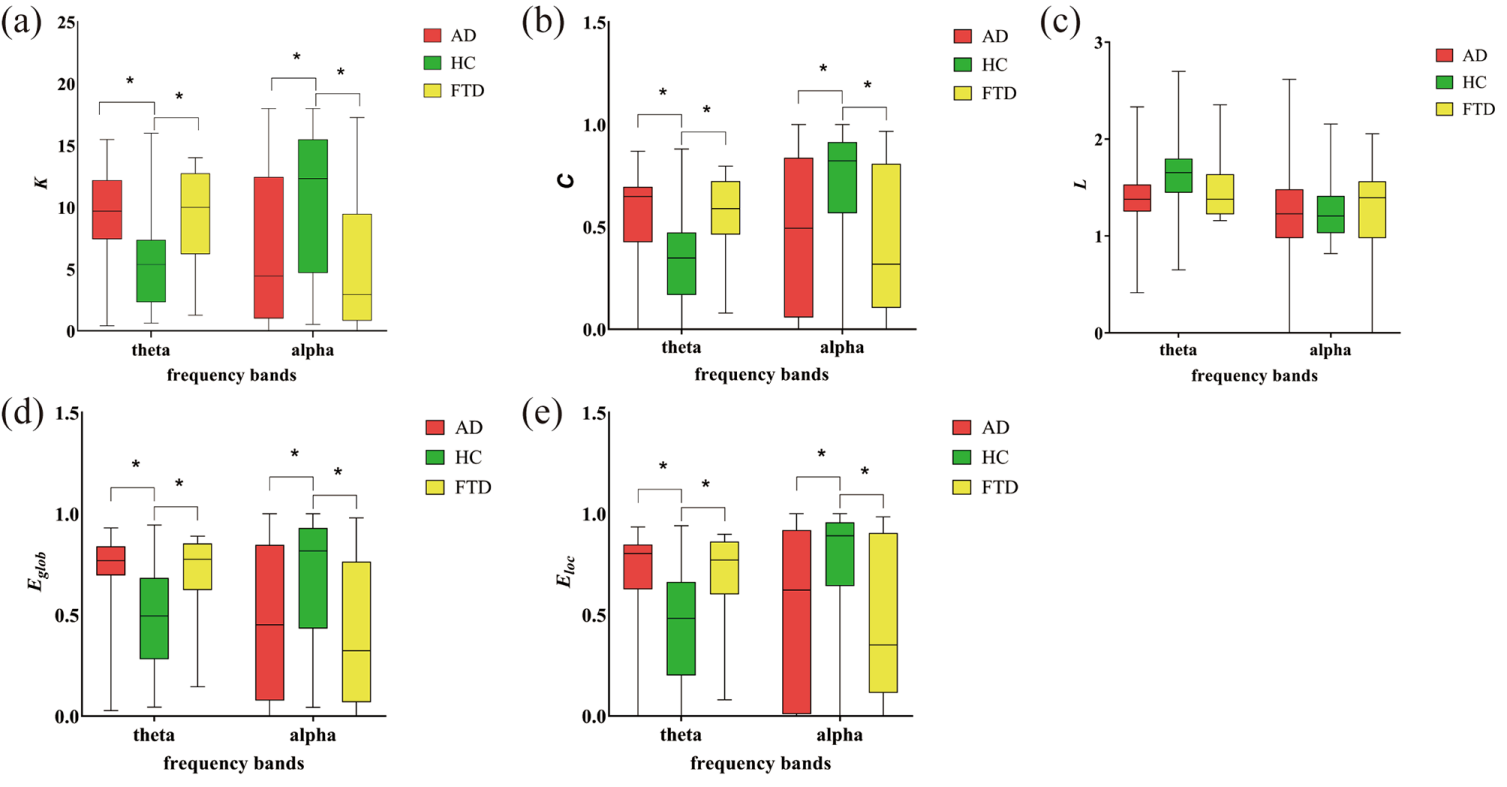
Graph-theoretic parameters of the brain network at theta and alpha bands. (**a**) Mean node degree. (**b**) clustering coefficient. (**c**) characteristic path length. (**d**) global efficiency. (**e**) local efficiency. The significant differences are denoted by the asterisk (corrected *p* < 0.05)

To quantify the metrics of the graph, graph-theoretic parameters of the brain network at theta and alpha frequency bands were calculated, depicted in Fig. 3; Table 2. Compared with the HC group, the $K$, $\text{C}$, ${E}_{glob}$, and ${E}_{loc}$ in the theta band are all significantly increased in the AD and FTD groups, whereas these parameters are significantly decreased in the alpha band. No significant differences in L were found between the three groups.

**Table 2:** Graph-theoretic parameters of brain network in AD, HC, and FTD patients at theta and alpha frequency bands. Parameters were pairwise compared between AD, HC, and FTD using permutation testing (*p* < 0.05). The p-values were corrected for multiple comparisons by the Bonferroni correction. The values in parentheses are mean values of Parameters. Significant differences are given in bold

Parameters	Frequency bands	P values (three groups)	P values (Pairwise comparisons)		
			AD/HC	FTD/HC	AD/FTD
**K**	theta	**0.005**	**0.003(9.149/5.652)**	**0.010(9.199/5.652)**	1.000(9.149/9.199)
	alpha	**0.003**	**0.011(6.278/10.679)**	**0.005(5.451/10.679)**	1.000(6.278/5.451)
**C**	theta	**0.007**	**0.005(0.551/0.355)**	**0.012(0.549/0.355)**	1.000(0.551/0.549)
	alpha	**0.005**	**0.008(0.448/0.712)**	**0.004(0.418/0.712)**	1.000(0.448/0.418)
**L**	theta	0.086	0.098(1.437/1.646)	0.311(1.472/1.646)	1.000(1.437/1.472)
	alpha	0.840	1.000(1.199/1.278)	1.000(1.209/1.178)	1.000(1.199/1.209)
**Eglob**	theta	**0.004**	**0.003(0.706/0.509)**	**0.010(0.706/0.509)**	1.000(0.706/0.706)
	alpha	**0.004**	**0.016(0.472/0.710)**	**0.005(0.409/0.710)**	1.000(0.472/0.409)
**Eloc**	theta	**0.005**	**0.004(0.684/0.448)**	**0.007(0.689/0.448)**	1.000(0.684/0.689)
	alpha	**0.007**	**0.011(0.499/0.769)**	**0.005(0.465/0.769)**	1.000(0.499/0.465)

### Graph analysis properties of different brain regions at the theta and alpha frequency bands

**Fig. 4:**
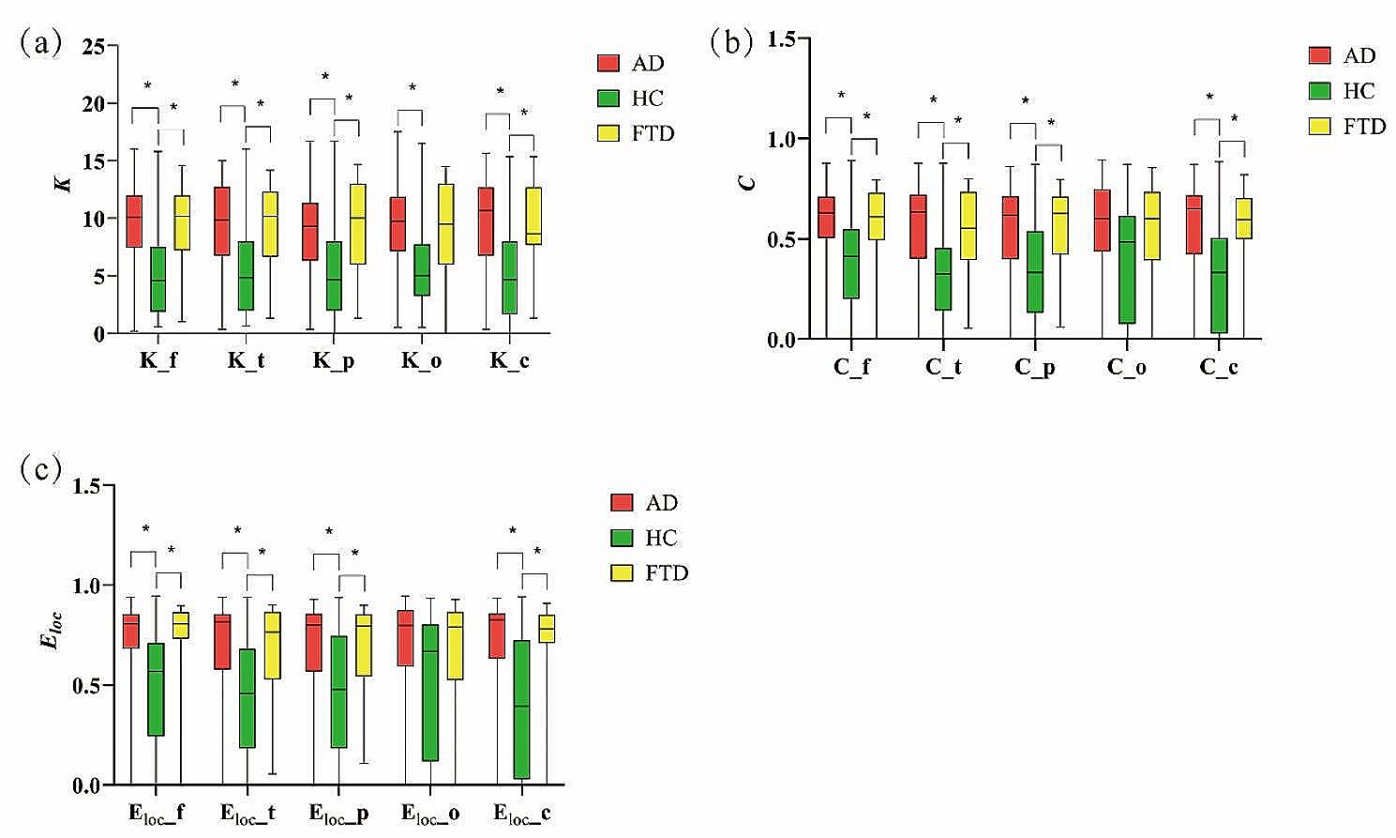
The graph analysis properties of different brain regions in theta frequency band. K_f is the mean node degree of frontal. K_t is the mean node degree of temporal. K_p is the mean node degree of parietal. K_o is the mean node degree of occipital. K_c is the mean node degree of central. C_f is the clustering coefficient of frontal. C_t is the clustering coefficient of temporal. C_p is the clustering coefficient of parietal. C_o is the clustering coefficient of occipital. C_c is the clustering coefficient of central. Eloc_f is the local efficiency of frontal. Eloc_t is the local efficiency of temporal. Eloc_p is the local efficiency of parietal. Eloc_o is the local efficiency of occipital. Eloc_c is the local efficiency of central. The significant differences are denoted by the asterisk (corrected *p* < 0.05)

**Fig. 5:**
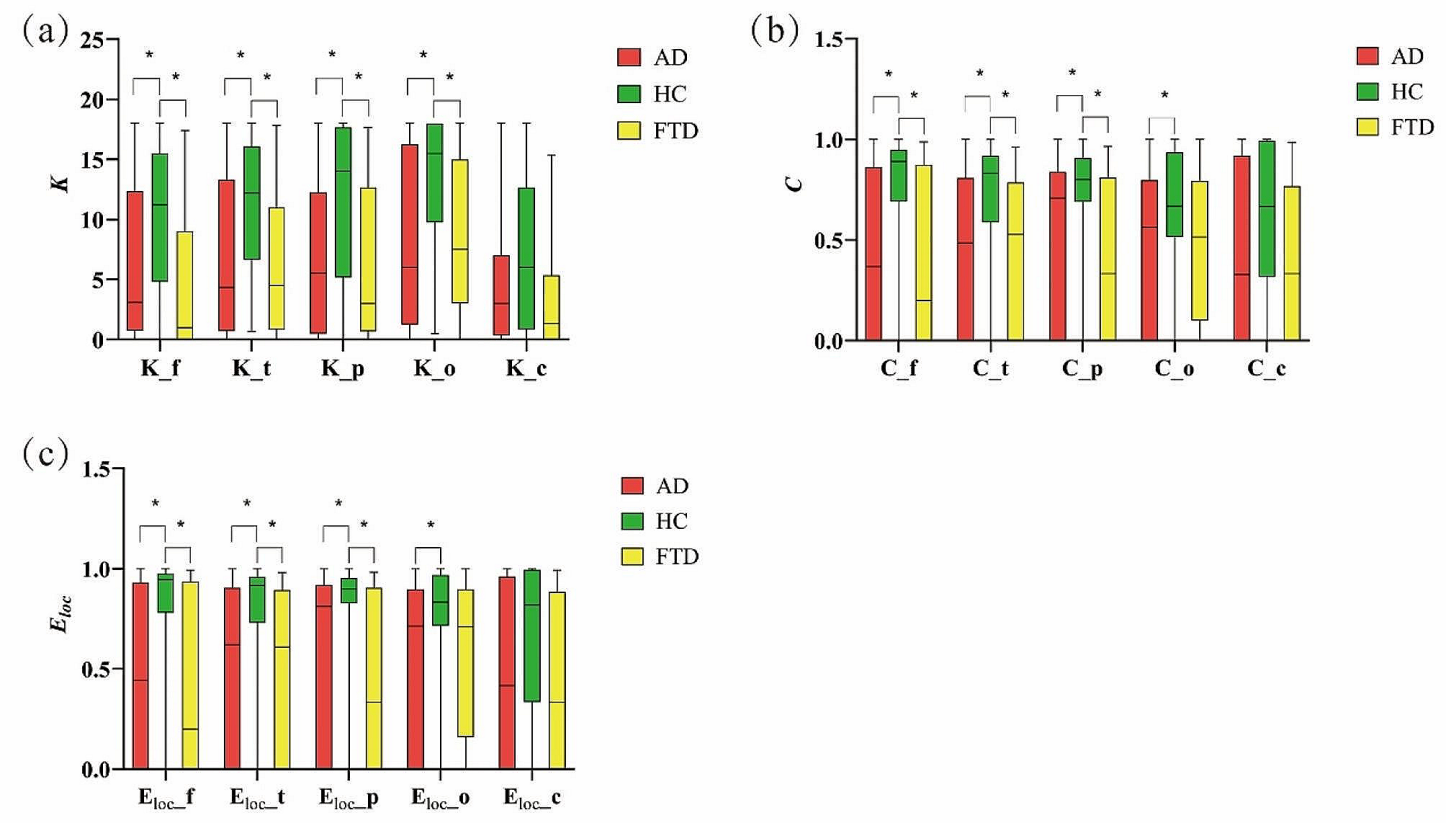
The graph analysis properties of different brain regions in alpha frequency band. K_f is the mean node degree of frontal. K_t is the mean node degree of temporal. K_p is the mean node degree of parietal. K_o is the mean node degree of occipital. K_c is the mean node degree of central. C_f is the clustering coefficient of frontal. C_t is the clustering coefficient of temporal. C_p is the clustering coefficient of parietal. C_o is the clustering coefficient of occipital. C_c is the clustering coefficient of central. Eloc_f is the local efficiency of frontal. Eloc_t is the local efficiency of temporal. Eloc_p is the local efficiency of parietal. Eloc_o is the local efficiency of occipital. Eloc_c is the local efficiency of central. The significant differences are denoted by the asterisk (corrected *p* < 0.05)

Further, we explored the graph analysis properties of different brain regions at the theta and alpha frequency bands. Nineteen channels were divided into 5 regions according to the location of electrodes, including frontal (Fp1, Fp2, F3, F4, Fz), temporal (T3, T4, T5, T6, F7, F8), parietal (P3, Pz, P4), occipital (O1, O2), and central (C3, Cz, C4). The mean node degree, clustering coefficient, and local efficiency of these five brain regions were calculated for AD, HC, and FTD patients in the theta band, with results shown in Fig. 4 and detailed in Table 3. For the alpha band, these metrics were also computed, as depicted in Fig. 5 and detailed in Table 4.

Our findings indicate that, in the theta band, patients with AD and FTD both exhibited significantly increased $K$, $\it \text{C}$, and ${E}_{loc}$ in the frontal, temporal, parietal, and central regions compared to HC. For the occipital region, a significant decrease in $\it \text{K}$ was observed exclusively in AD patients relative to the HC. In the alpha band, we observed a pronounced reduction in $\it \text{K}$, $\it \text{C}$, and ${E}_{loc}$ within the frontal, temporal, and parietal regions for both AD and FTD patients. However, in the parietal region, the decrease in $C$, and ${E}_{loc}$ was significant only for the AD group, with no corresponding alterations observed in the FTD group. Notably, no statistically significant differences were detected in the central region across all groups.

**Table 3:** Graph analysis properties of different brain regions in theta frequency band. Parameters were pairwise compared between AD, HC, and FTD using permutation testing (*p* < 0.05). The p-values were corrected for multiple comparisons by the Bonferroni correction. The values in parentheses are mean values of Parameters. Significant differences are given in bold

Parameters	*P* values (three groups)	*P* values (Pairwise comparisons)		
		AD/HC	FTD/HC	AD/FTD
**K_f**	**0.003**	**0.003(9.150/5.441)**	**0.006(9.287/5.441)**	1.000(9.150/9.287)
**K_t**	**0.009**	**0.006(9.227/5.770)**	**0.018(9.203/5.770)**	1.000(9.227/9.203)
**K_p**	**0.013**	**0.028(8.620/5.805)**	**0.031(9.058/5.805)**	1.000(8.620/9.058)
**K_o**	**0.013**	**0.006(9.222/5.914)**	0.099(8.587/5.914)	1.000(9.222/8.587)
**K_c**	**0.001**	**0.001(9.472/5.437)**	**0.003(9.594/5.437)**	1.000(9.472/9.594)
**C_f**	**0.018**	**0.031(0.551/0.384)**	**0.028(0.565/0.384)**	1.000(0.551/0.565)
**C_t**	**0.003**	**0.002(0.549/0.327)**	**0.009(0.534/0.327)**	1.000(0.549/0.534)
**C_p**	**0.010**	**0.014(0.545/0.356)**	**0.018(0.553/0.356)**	1.000(0.545/0.553)
**C_o**	0.117	0.116(0.541/0.392)	0.202(0.541/0.692)	1.000(0.541/0.541)
**C_c**	**0.003**	**0.002(0.567/0.336)**	**0.014(0.554/0.336)**	1.000(0.567/0.554)
**E**_**loc**_ **_f**	**0.009**	**0.017(0.686/0.477)**	**0.016(0.705/0.477)**	1.000(0.686/0.705)
**E**_**loc**_ **_t**	**0.002**	**0.001(0.683/0.416)**	**0.005(0.676/0.416)**	1.000(0.683/0.676)
**E**_**loc**_ **_p**	**0.009**	**0.009(0.678/0.451)**	**0.018(0.682/0.451)**	1.000(0.678/0.682)
**E**_**loc**_ **_o**	0.099	0.108(0.671/0.495)	0.181(0.674/0.495)	1.000(0.671/0.674)
**E**_**loc**_ **_c**	**0.002**	**0.002(0.701/0.423)**	**0.006(0.705/0.423)**	1.000(0.701/0.705)

**Table 4:** Graph analysis properties of different brain regions in alpha frequency band. Parameters were pairwise compared between AD, HC, and FTD using permutation testing (*p* < 0.05). The p-values were corrected for multiple comparisons by the Bonferroni correction. The values in parentheses are mean values of Parameters. Significant differences are given in bold

Parameters	*P* values (three groups)	*P* values (Pairwise comparisons)		
		AD/HC	FTD/HC	AD/FTD
**K_f**	**< 0.001**	**0.023(5.933/10.083)**	**0.002(4.122/10.083)**	0.724(5.933/4.122)
**K_t**	**0.008**	**0.014(6.907/11.448)**	**0.010(6.355/11.448)**	1.000(6.907/6.355)
**K_p**	**0.003**	**0.005(6.704/12)**	**0.006(6.087/12)**	1.000(6.704/6.087)
**K_o**	**0.005**	**0.003(7.819/13.466)**	**0.009(8.217/13.466)**	1.000(7.819/8.217)
**K_c**	**0.025**	0.102(4.139/6.954)	0.066(3.377/6.954)	1.000(4.139/3.377)
**C_f**	**0.002**	**0.004(0.427/0.746)**	**0.002(0.375/0.746)**	1.000(0.427/0.375)
**C_t**	**0.010**	**0.006(0.442/0.723)**	**0.017(0.460/0.723)**	1.000(0.442/0.460)
**C_p**	**< 0.001**	**0.012(0.502/0.755)**	**0.001(0.411/0.755)**	1.000(0.502/0.411)
**C_o**	0.054	**0.043(0.472/0.680)**	0.063(0.481/0.680)	1.000(0.472/0.481)
**C_c**	0.078	0.217(0.423/0.609)	0.109(0.373/0.609)	1.000(0.472/0.481)
**E**_**loc**_ **_f**	**0.001**	**0.004(0.467/0.792)**	**0.001(0.398/0.792)**	1.000(0.467/0.398)
**E**_**loc**_ **_t**	**0.018**	**0.008(0.502/0.786)**	**0.024(0.518/0.786)**	1.000(0.502/0.518)
**E**_**loc**_ **_p**	**0.002**	**0.013(0.555/0.824)**	**< 0.001(0.469/0.824)**	1.000(0.555/0.469)
**E**_**loc**_ **_o**	**0.045**	**0.034(0.553/0.781)**	0.070(0.585/0.781)	1.000(0.553/0.585)
**E**_**loc**_ **_c**	0.066	0.231(0.450/0.636)	0.088(0.385/0.636)	1.000(0.450/0.385)

## Discussion

In this study, we aimed to explore the alterations in brain network connectivity and topological characteristics in patients with AD and FTD, as well as in comparison to a group of HC. Guided by a previous AD research [7], we applied the PLI to measure functional connectivity in the lower frequency bands (delta and theta) and the AEC-c to evaluate connectivity in the higher frequency bands (alpha, beta, and gamma), which minimizes biases arising from volume conduction and activity in common sources, such as reference effects. Subsequently, we conducted a graph-theoretical analysis to scrutinize the topological variations across the brain networks of these three distinct groups.

Our results indicated that AD and FTD patients exhibited the same abnormal patterns in whole-brain functional connectivity values and topological properties. Both AD and FTD patients showed a significant enhancement of brain network connectivity in the theta band, characterized by a significant increase in the mean whole-brain PLI, $\it \text{K}$, $\it \text{C}$, ${E}_{glob}$, and ${E}_{loc}$. In contrast, in the alpha band, there was a significant weakening of brain network connectivity, characterized by a significant decrease in the mean whole-brain AEC-c, $\it \text{K}$, $\it \text{C}$, ${E}_{glob}$, and ${E}_{loc}$.

In the analysis of network topological properties across different brain regions, both AD and FTD patients exhibited significant increases in $\it \text{K}$, $\it \text{C}$, ${E}_{loc}$in the frontal, temporal, and parietal regions in the theta band. Conversely, in the alpha band, both patient groups showed significant decreases in $\it \text{K}$, $\it \text{C}$, ${E}_{loc}$in these same regions. This suggests that, in both the theta and alpha bands, the frontal, temporal, and parietal regions are implicated in abnormal network connectivity for both AD and FTD patients. However, in the central region, patients with AD and FTD only exhibited abnormal network connectivity in the theta band, with no significant changes found in the alpha band.

Interestingly, in the occipital region, patients with AD and FTD exhibited distinct patterns of abnormal brain network connectivity. Specifically, in AD patients, the $\it \text{K}$ in the theta band was significantly higher than in HC, whereas the $\it \text{C}$ and ${E}_{loc}$ in the alpha band were significantly lower than those in HC. In contrast, no significant changes were observed in FTD patients.

We have compared our results with previous findings in literature. The increase in whole-brain PLI in the theta band of AD patients is consistent with some previous studies [7, 45, 46]. This finding might indicate a compensatory mechanism where the brain attempts to maintain cognitive function despite the pathological changes associated with AD [7]. Additionally, the decrease of AEC-c in the alpha band is also supported by previous MEG and EEG studies [47, 48]. This, along with the observed decrease in both global and local efficiency of the brain, appears to confirm that AD is often characterized as a ‘disconnection syndrome’, which is marked by a loss of network integrity and altered synchronizability in the higher-frequency bands [3, 22, 49].

Several studies have reported that the EEG in FTD tends to remain normal or show only minor deviations until late in the course of the disease [50, 51]. In our study, we observed that patients with FTD demonstrate an enhancement of functional connectivity in the theta band and a weakening in the alpha band, similar to patterns seen in AD. This discrepancy can be explained by the use of different functional connectivity measures. The PLI and AEC-c are both measures that are less sensitive to the effects of volume conduction, making them more reproducible and valid [7]. Therefore, our results are less affected by spurious estimates of interactions.

Although there is considerable clinical interest in the differential diagnosis of AD and FTD, few studies have directly compared the two dementias. Our study revealed that patients with AD and FTD exhibit distinct brain network connectivity and topological characteristics. Patients with AD showed more extensive and severe abnormalities in brain network connectivity, with effects observed across all brain regions. However, those with FTD exhibited relatively preserved function in the occipital region, which is primarily responsible for processing visual information [52]. Notably, in AD patients, the mean node degree in the occipital region significantly increased in the theta band, while the clustering coefficient and local efficiency notably decreased in the alpha band. However, these significant changes were not observed in FTD patients. Given that visual spatial dysfunction is characteristic of AD and not typically seen in FTD [53, 54], the topological parameters of the occipital region may serve as valuable electrophysiological markers for distinguishing between AD and FTD.

Although our study yielded significant results, it is not without limitations. Firstly, our research was conducted with a relatively small subject sample. Future studies should aim for larger sample sizes to validate our findings. Secondly, while our study focused on AD and FTD, there are various dementia subtypes, such as vascular dementia (VaD) and dementia with Lewy bodies (DLB), which often lead to clinical confusion. It is crucial to include a broader range of dementia subtypes in future research. Thirdly, our cross-sectional design limits our ability to track changes in brain network properties over time. Therefore, longitudinal studies should be undertaken to monitor disease progression. Fourthly, despite the existence of over 15 brain network topology parameters [44], we only examined the five most common ones. A comprehensive analysis of all available topology parameters should be considered in subsequent studies. Lastly, while some studies have shown the potential of modulating gamma oscillations for treating central nervous system diseases [55, 56], our study did not reveal significant differences in the gamma band among the three groups. One possible explanation is the absence of cognitive tasks specific to AD or FTD in our design. Future research should incorporate task-specific assessments of gamma oscillations.

## Conclusions

Overall, we found that the brain network connectivity and topological characteristics of AD and FTD patients both exhibit different rhythmic characteristics in different frequency bands, with enhanced functional connectivity in the theta band and diminished function in the alpha band. Our findings contribute to understanding of the pathological mechanisms of AD and FTD at the level of brain networks, as well as how these diseases affect the functional coordination of different brain regions. Furthermore, patients with AD showed a loss of function across the whole brain, while patients with FTD retained the function in the occipital region, which may provide new insights for the development electrophysiological markers for the clinical diagnosis of AD and FTD.

## Data Availability

The following publicly available dataset was analyzed in this work: Andreas Miltiadous and Katerina D. Tzimourta and Theodora Afrantou and Panagiotis Ioannidis and Nikolaos Grigoriadis and Dimitrios G. Tsalikakis and Pantelis Angelidis and Markos G. Tsipouras and Evripidis Glavas and Nikolaos Giannakeas and Alexandros T. Tzallas (2023). A dataset of EEG recordings from: Alzheimer’s disease, Frontotemporal dementia and Healthy subjects. OpenNeuro. [Dataset] doi: doi: https://doi.org/10.18112/openneuro.ds004504.v1.0.6.

## Abbreviations

AD: Alzheimer’s disease
FTD: Frontotemporal dementia
EEG: Electroencephalogram
HC: Healthy controls
PLI: Phase Lag Index
AEC-c: Amplitude Envelope Correlation with leakage correction
MEG: Magnetoencephalography
fMRI: Functional magnetic resonance imaging
DSM: Diagnostic and Statistical Manual of Mental Disorders
ICD: International Classification of Diseases
ICA: Independent Component Analysis
AEC: Amplitude envelope correlation
MMSE: Mini-Mental State Examination
IQR: Interquartile range
VaD: Vascular dementia
DLB: Dementia with Lewy bodies
